# Does Entrepreneurs’ Darwinian Social Identity Contribute to Business Performance via Corporate Social Responsibility in China? The Role of Entrepreneurs’ Well-Being

**DOI:** 10.3389/fpsyg.2021.781399

**Published:** 2021-12-14

**Authors:** Jinliang Chen, Ning Chris Chen, Kangkang Yu, Colin Michael Hall

**Affiliations:** ^1^Business School, Central University of Finance and Economics, Beijing, China; ^2^Department of Management, Marketing, and Entrepreneurship, University of Canterbury, Christchurch, New Zealand; ^3^School of Agricultural Economics and Rural Development, Renmin University of China, Beijing, China

**Keywords:** social identity, corporate social responsibility, business performance, entrepreneurs’ well-being, stakeholder theory

## Abstract

Although the impact of entrepreneurs’ social identity on successful entrepreneurship has attracted much scholarly attention, it is often to evaluate successful entrepreneurship through direct channel to financial performance. Recently, there is a growing body of researches beginning to pay attention to the impact of entrepreneurs’ social identity on corporate social responsibility (CSR) regarded as indirect social aspect channel to successful entrepreneurship. However, little is known regarding how entrepreneurs’ Darwinian social identity affects CSR, which in turn, affects business performance. This study addresses this issue by combining stakeholder theory with social identity theory, to investigate the relationship between entrepreneurs’ Darwinian social identity and business performance via CSR. In addition, the moderating effect of entrepreneur’s well-being is further examined to uncover the interaction effect of the individual psychological resource on business performance. The empirical results indicate that entrepreneurs’ Darwinian social identity contributes positively to CSR, so as further to business performance. In addition, this relationship is further found to be significantly moderated by entrepreneurs’ well-being. The results indicate that entrepreneurs can achieve business success via CSR, by which entrepreneurs can further acquire successful entrepreneurship through caring more about their well-being.

## Introduction

Recently, the relationship between entrepreneurs’ social identity and successful entrepreneurship has attracted considerable scholarly attention within the entrepreneurship and social psychology domains. That is, entrepreneurs’ behaviors are perceived to evolve with the firm’s development because individual identities of entrepreneurs are regarded as being expressed and manifested in business activities (de la Cruz et al., 2018). In particular, entrepreneurs define themselves through their own understanding of “who I am” and “who I want to be” and position themselves in relation to particular social groups. Furthermore, this process cultivates their social values and norms; thus, guiding the interpretation of their business role identity of “what it means to be an entrepreneur” (Powell and Baker, 2014; Zuzul and Tripsas, 2020).

Previous literature explored entrepreneurs’ social identities and their influence on business performance on the basis of social identity theory from the field of social psychology (Ashforth and Mael, 1989; Hoggy and Terry, 2000) and environmental psychology (Chen et al., 2021). Notably, the distinct types of entrepreneurs’ social identity reflect their levels of passion for particular business actions, such as growing a venture (Cardon et al., 2009) or launching new firms (Fauchart and Gruber, 2011). Numerous studies have investigated the role of the distinct types of entrepreneurs’ social identity (Fauchart and Gruber, 2011; Sieger et al., 2016). Among them, Darwinian, communitarian, and missionary social identity are three key levels of entrepreneurs’ social identity, to illustrate the levels of social inclusiveness in founders’ social identities. Entrepreneurs’ Darwinian social identity is the fundamental level of entrepreneurs’ social identity, and reflects an entrepreneur’s pursue for business survival and success, which refers to the common goal of any business (Sieger et al., 2016). Regarded as the most generic type of business social identity, Darwinian social identity applies to most businesses in a society. In contrast, communitarian and missionary social identities, respectively, reflecting the aim of solving a specific problem for a specific community or the society in general, are particularly studied in contexts of social entrepreneurship. Therefore, Entrepreneurs’ Darwinian social identity is the first layer of entrepreneurs’ social identity, and thus valuable in understanding its influences on variables at the corporate level.

In reviewing the current scholarly studies, two research gaps remain regarding entrepreneurs’ Darwinian social identity. First, the current investigations have focused on the role of entrepreneurs’ Darwinian social identity from the direct effect to financial performance but relatively ignored the indirect effect through social aspects. The goal of entrepreneurs’ Darwinian social identity is to pursue success not only by acquiring profit performance but also by obtaining the improvement of social aspects (Fauchart and Gruber, 2011). However, previous studies relatively neglected the social aspects improvement of entrepreneurs’ Darwinian social identity to achieve a successful business (de la Cruz et al., 2018). Second, studies divulging the interaction effect of the individual psychological resource with entrepreneurs’ social identity on firm outcomes are limited. Recently, entrepreneurs’ well-being, the degree to which people are content with their lives and jobs (Deng et al., 2019), has been regarded as a key psychological resource in the entrepreneurship area, as it intends to complement traditional outcomes of entrepreneurial activities, such as firm performance (Wiklund et al., 2019). Although entrepreneurs’ well-being is considered a critical psychological resource related to the creation, production, and cooperation (see Wiklund et al. (2019)], its interaction role with entrepreneurs’ social identity on firm outcomes is rarely investigated.

Corporate social responsibility (CSR) illustrates the process by which corporations integrate business ethics into their operations, adapt and socialize themselves into society, gain a social license to operate, and eventually become a “corporate social citizen.” According to the previous studies, upper echelons theory point out the crucial role of certain individuals in a firm, such as the owners and top managers, which may be deemed significant for CSR (Hambrick and Mason, 1984; Ponzi et al., 2011; Cahan et al., 2015). In addition, CSR has a significant influence on firms’ reputations in society and furthers their ability to survive and thrive in the contemporary business environment. In other words, the importance of CSR in the contemporary business environment has been well-recognized (Burke and Logsdon, 1996; Snider et al., 2003; Bolourian et al., 2021; Gillan et al., 2021). Considering the increased concern surrounding the dominance of material pursuits in Chinese business practice (Ni and Davidson, 2021), developing an in-depth understanding of the role of entrepreneurs on CSR in business development in China is therefore crucial, including how CSR and business performance relate to each other.

Entrepreneurs’ well-being, defined as “the experience of satisfaction, positive affect, infrequent negative affect, and psychological functioning in relation to developing, starting, growing, and running an entrepreneurial venture” (Wiklund et al., 2019, p. 582), also has attracted a significant amount of scholarly attentions. Well-being is a term adopted in many disciplines (Ryan and Deci, 2001), which has been described as “living in a state that is in some sense good” (Warr, 2013, p. 77) and is characterized by “optimal psychological functioning and experience” (Ryan and Deci, 2001, p. 142). For example, work-related subjective well-being explains the degree to which people are satisfied with their jobs (Deng et al., 2019). Regarding entrepreneur’s well-being, a typical of well-being in entrepreneurship, it has studied as an outcomes traditionally. For instance, the role of entrepreneurs’ traits on entrepreneur’s well-being has been extensively examined in previous studies (Shir et al., 2019; Nikolaev et al., 2020; Amorós et al., 2021). Recent years, entrepreneur’s well-being has been thought to be an antecedent as a critical psychological resource in entrepreneurship research (Wiklund et al., 2019). However, to our knowledge, a lot remains unknown regarding the functioning of entrepreneur’s well-being as a psychological resource on the effect of entrepreneurs’ social identity.

Keeping the abovementioned aspects in mind, in this study, we aim to fill the research gaps by exploring the role of entrepreneur’s Darwinian social identity in Chinese context. This study examines its effects on CSR and business performance, while considering the moderating effect of the entrepreneur’s well-being. According to social identity theory, identities are the main drivers of the individuals’ behavior associated with their business roles, such as directors or chief executive officers (Murnieks and Mosakowski, 2007; Zuzul and Tripsas, 2020). The manifestation of social identity in business actions is prominent when the business role of an individual is an entrepreneur or founder (Murnieks et al., 2014; Wry and York, 2017; Grimes, 2018). In addition, according to stakeholder theory, organizations should pay attention to long-term sustainable and social responsibility issues, rather than short-term financial profits (Barnett, 2007; Turker, 2009). As such, this study explores the mediating role of CSR between entrepreneur’s Darwinian social identity and business performance, as well as the moderating effect of the entrepreneur’s well-being by combing the social identity theory and stakeholder theory with theory integration (Suddaby et al., 2011; Mayer and Sparrowe, 2013).

Investigating the above research gaps suggests four possible theoretical contributions. First, this study contributes to the theoretical logic underlying the relationship between entrepreneurs’ Darwinian social identity and business performance. The present study indicates that CSR is a significant channel that entrepreneurs’ Darwinian social identity influences business performance, which enriches its underlying non-financial mechanism. Second, this present study adds to social identity theory by introducing a broaden-and-build perspective. With empirically investigating the moderating effect of entrepreneurs’ well-being on the link between entrepreneurs’ Darwinian identity and business performance via CSR, this study enriches social identity theory from a broaden-and-build perspective. Third, this study contribute to the literature of theory integration. The present study combines social identity theory and stakeholder theory together to disclose the mechanism between entrepreneurs’ Darwinian social identity and business performance via through CSR, which provides a combining mode for theory integration theory. Fourth, investigating the linkage between entrepreneurs’ Darwinian social identity and CSR provides a perspective to examine the extension of entrepreneurs’ selves to corporation entities, and to trace the drivers of “corporate citizenship” to entrepreneurs’ characteristics, personalities, and identities, linking social identity theory and corporate citizenship theory.

This paper is further organized as follows: First, social identity theory and stakeholder theory are reviewed along with CSR to develop hypotheses and conceptualize the theoretical model. Second, the empirical research approach is explained based on a quantitative survey of business owners and entrepreneurs in China. Third, data analysis is reported in detail, followed by discussion and findings.

## Theoretical Development

### Social Identity Theory

Based on social identity theory, Fauchart and Gruber (2011) proposed three key levels of social identity from an entrepreneur perspective, namely, “Darwinian social identity,” “communitarian social identity,” and “missionary social identity,” to reflect an individual’s social identity and relationships in terms of “personal and symbolic interaction with others and level of social inclusion” (de la Cruz et al., 2018, p. 91). In particular, when driven by Darwinian social identity, entrepreneurs tend to benchmark themselves against their competitors and other entrepreneurs in the general business environment to evaluate their own success. Their primary goal is to create a strong, profitable, and thus surviving business, which is naturally evaluated through business performance (Fauchart and Gruber, 2011; de la Cruz et al., 2018). In contrast, communitarian social identity focuses on belonging to a social group and thus serving a community (Lewis, 2013). A communitarian identity-drives firm in valuing its ability to contribute to the community through its business and products (Fauchart and Gruber, 2011; Hall and Williams, 2020). Missionary social identity is geared toward causes and political goals; it often motivates innovation and creative solutions with a political mission rather than competitive advantage in certain industries (Fauchart and Gruber, 2011).

The most generic type of business social identity is Darwinian social identity, which is defined as aiming to be a strong and successful business, reflecting the “classic” businessperson image (Van Praag, 1999). Notably, Social Darwinism is a misreading of Darwinian evolutionary theory in terms of social ecology, which nevertheless remains a commonly applied concept (Caudill, 2005). However, the notion of Darwinian used in this study is an interpretation of the application of the notion of “survival of the fittest.” It acts as a key driver of almost any entrepreneurial activity, for this survival nature. By contrast, both communitarian social identity and missionary social identity are less common than Darwinian social identity, and mostly find their applications in social entrepreneurship for their implications for social goals (Wry and York, 2017; Ko and Kim, 2020). Specifically, missionary social identity has significant implications in business innovations with a political mission (Smith and Woodworth, 2012).

Social identity theory helps explain the heterogeneity of business actions in the process of starting up a new firm, with entrepreneurs’ Darwinian social identity influencing business performance directly and through effectuation (de la Cruz et al., 2018), internal and external audience expectation (Zuzul and Tripsas, 2020), and nascent entrepreneurial behaviors (Seibert et al., 2021). The main reason is that entrepreneurs’ Darwinian social identity shapes their priorities for their businesses and strategies (Miller and Breton-Miller, 2011). Moreover, such entrepreneurs’ Darwinian social identity is deemed vital in the decision-making of the firm development, including innovation (Smith and Woodworth, 2012), social orientations (Pan et al., 2019; Ko and Kim, 2020), and any decisions made under uncertainty or to eliminate uncertainty (Alsos et al., 2016). Based on this reasoning, this study proposes the following hypothesis:

*H1. Entrepreneurs’ Darwinian social identity has a positive effect on business performance.*

### Mediating Effect of CSR

Corporate social responsibility arises because of the need for businesses to take measures that will reflect broader ethical considerations of their actions, including labor conflict, issues with mass production, and other social problems (Rodriguez-Gomez et al., 2020). CSR has become increasingly significant to business and business researchers in recent decades [see the review of Ye et al. (2021)]. As more public attention is paid to CSR, firm decision-makers are faced with the problem of how to manage social and community engagement and how to evenly allocate their limited resources among competing priorities. A widely cited approach to the scope of CSR proposed by Carroll (1979) identifies four categories of obligations that the business has to society, ranging in a relative proportion of economic responsibilities to legal, ethical, and discretionary responsibilities in relationships with different stakeholders. Barnett (2007) argued that stakeholder relationship orientation is an essential but often implicit characteristic of the business case for CSR. He defined CSR as “a discretionary allocation of corporate resources to improving social welfare that serves as means of enhancing relationships with key stakeholders” (p. 801). Thus, CSR can be defined as corporate behaviors that aim to positively affect stakeholders and go beyond its economic interest to meet the need for social, economic, and environmental sustainability (Turker, 2009; Freudenreich et al., 2020; Mio et al., 2020).

According to social identity theory, any entrepreneurial activity is an outcome of the externalization process of entrepreneurs’ social identification and categorization, which then provides a fundamental frame of reference of how they perceive themselves relative to others and society (Alsos et al., 2016). This implies an association of CSR, with Darwinian, communitarian, and missionary social identities, respectively, since they reveal the three levels of entrepreneurs’ social identity. From a Darwinian social identity perspective specifically, entrepreneurs tend to benchmark themselves against their competitors and other entrepreneurs in the business environment in evaluating their own success. The primary goal of creating a successful business is both through financial performance and through social output (Fauchart and Gruber, 2011; de la Cruz et al., 2018). Financial performance improvement is the direct channel to achieve business performance, whereas social output such as CSR is the indirect channel to realize successful business. With the increasing importance of CSR in the sustainable development, the indirect effect of CSR is necessary to be investigated between entrepreneurs’ Darwinian social identity and business performance. Following the underlying logic of stakeholder theory, organizations should move their attention from short-term financial profits to long-term sustainable and social responsibility issues to share more value with stakeholders (Barnett, 2007; Turker, 2009). With the integration of social identity theory and stakeholder theory following the logic of theory integration theory, the mediating effect of CSR is investigated here to reveal the indirect channel between entrepreneurs’ Darwinian social identity and business performance (Suddaby et al., 2011; Mayer and Sparrowe, 2013).

Following this logic, an entrepreneur’s Darwinian social identity implies the entrepreneur’s desire of a successful business entity to extend the entrepreneurial self into society. As such, besides the direct channel to business performance, an entrepreneurs’ Darwinian social identity is also indicative of defining a business in terms of its social position and reputation, of which, CSR is potentially a major component. In particular, CSR can become an important component of a firm’s value of common equity, which influences how successfully a firm is socially evaluated (Malik, 2015). Based on this logic, a hypothesis is proposed to indicate the relationship between entrepreneurs’ Darwinian social identity and CSR:

*H2a. Entrepreneurs’ Darwinian social identity has a positive effect on CSR.*

The number of empirical studies focusing on the impacts of CSR has grown substantially since the 1980s. Some of these studies have attempted to identify how CSR affects business performance and provided empirical evidence on the relationship between corporate behaviors and organizational reputation, competitiveness, and sustainability (Burke and Logsdon, 1996; Porter and Kramer, 2002; Johnson, 2003; Snider et al., 2003). Numerous studies identified that CSR is often an antecedent of financial performance (Margolis and Walsh, 2003; Orlitzky et al., 2003; Allouche and Laroche, 2005; Van Beurden and Gossling, 2008; Moser and Martin, 2012; Velte, 2021). Furthermore, studies exploring this relationship mostly reported positive results (e.g., Van der Laan et al., 2008; Wang et al., 2016).

Stakeholder theory suggests that firms satisfying social expectations through implicit social contracts would minimize the costs of maintaining relationships with stakeholders and obtain higher financial returns (Jones, 1995; Swanson, 1995). Preston and O’Bannon (1997) developed a social influence proposition that a high level of CSR would meet the demands of a wide range of stakeholders, thereby positively affecting financial performance. Moreover, firms with good CSR performance are expected to have better business credit and market behaviors, which, in turn, will enhance their brand reputation (Schuler and Cording, 2006). In addition, CSR practices perceived as improving product and service quality can enhance customer loyalty and willingness to repurchase (Galbreath and Shum, 2012; Saeidi et al., 2015; Xie et al., 2017). Overall, a wide range of stakeholders, including customers, employees, and investors, can be addressed through CSR activities and firm reward through productivity improvement, market recognition, and customer satisfaction, which all could contribute to better financial performance. As such, this study proposes the following hypothesis:

*H2b: CSR has a positive effect on business performance.*

### Moderating Effect of Entrepreneurs’ Well-Being

Entrepreneurs’ well-being, is not only an outcome, but also an antecedent. In particular, entrepreneurs’ well-being is a critical psychological resource for entrepreneurs to cope with stress when pursuing success, for that the work state of entrepreneurs can be extremely stressful because of uncertainty, complexity, and risk of failure, including time pressure and long work hours (Patzelt and Shepherd, 2011; Hahn et al., 2012; Uy et al., 2013; Cardon and Patel, 2015; Baron et al., 2016; Stephan, 2018). As such, entrepreneurs’ well-being is a significant psychological resource that influences the role of entrepreneurs’ Darwinian social identity on CSR. Furthermore, with the high position of an entrepreneur in a firm, entrepreneurs’ well-being as a psychological resource can influence the relationship between CSR and business performance.

Entrepreneurs with high levels of personal well-being can draw on both affective and cognitive resources in their work (Hobfoll, 2001). Entrepreneurs with affective resources are more satisfied with their own business, and such positive affection broadens their thoughts and helps them recognizes opportunities which, in turn, builds future resources (Fredrickson, 2001). This broaden-and-build perspective suggests that entrepreneurs with high well-being would pursue more opportunities to gain long-term benefits such as building corporate image and social responsibilities. In addition, entrepreneurs are engaged in their work with a high level of investment in cognitive resources. They have freedom over how to organize and schedule their tasks, and they can avoid the external control and restrictions in a hierarchical organization (Rindova et al., 2009; Stephan, 2018). This freedom provides them with autonomy to engage in meaningful work (Cardon et al., 2009; Baron et al., 2016), which also supports the adoption of strategies that focus on employees’ growth and capabilities (Benz and Frey, 2008; Bulmash, 2016). Hence, when entrepreneurs have a high level of well-being, they would likely have less intention to utilize Darwinian social identity to enhance CSR. As such, this study proposes the following hypothesis:

*H3a: Entrepreneurs’ well-being negatively moderates the effect of entrepreneurs’ Darwinian social identity on CSR.*

Entrepreneurs with a high level of well-being support the contribution of CSR to business performance because of the following three reasons: First, greater autonomy makes them more flexible and adaptive in adverse situations (Ryff, 2019), which reduces business risks and possibilities of failure. Second, a high need for success and achievement leads to high self-efficacy (Kolvereid and Bullvag, 1996; Bradley and Roberts, 2004), which refers to entrepreneurs’ belief in their ability to use cognitive resources and respond to environmental uncertainty (Stajkovic and Luthans, 1998; Nielsen et al., 2009). This stronger internal locus of control helps entrepreneurs accomplish important roles and tasks associated with CSR (McGee et al., 2009), for example, enhancing firm reputation, potentially leading to sustainable market growth, and competitive advantage. Third, entrepreneurs’ well-being also stems from effortful and self-determined activities (Ryan and Deci, 2001; Ryff, 2019), which increase engagement and commitment (Benz and Frey, 2008; Felfe and Heinitz, 2008). Leadership and social exchange attract talent and enhance employees’ loyalty to support operations and management, which, in turn, lead to better business performance. As such, this study proposes the following hypothesis:

*H3b: Entrepreneurs’ well-being positively moderates the effect of CSR on performance.*

**FIGURE 1:**
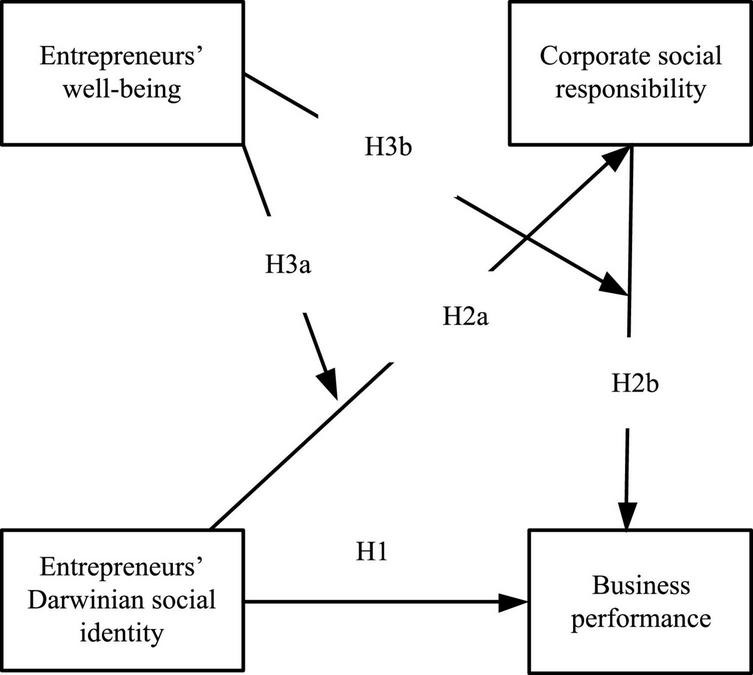
Conceptual model.

According to the above hypotheses, entrepreneurs’ Darwinian social identity positively affects CSR, which, in turn, enhances final business performance. Furthermore, these effects are contingent on entrepreneurs’ well-being, as shown in Figure 1.

## Materials and Methods

### Sample and Data Collection

In the Chinese entrepreneurial context, small and medium enterprises comprise the majority of the Chinese economy, accounting for 58.5% of GDP in 2009 (Zhou, 2012). Chinese entrepreneurs are mainly driven by profit and commercial value within the ambiguous rules and limited institutional framework of business governance in China (Cinar et al., 2018). Accordingly, Darwinian social identity dominates the Chinese entrepreneurs’ mentality (Kong, 2017; Su et al., 2020). Therefore, this study has focused on this generic level of business social identity, that is, entrepreneurs’ Darwinian social identity, to capture the majority of entrepreneurs in the Chinese business context.

A larger-scale questionnaire survey was conducted in the Shandong province of China in June, July, and August 2021 to collect data to test the theoretical model. Shandong province has been identified to be an ideal place for this study for several reasons. First, located in China’s eastern coastal areas where connecting the capital area and Yangtze River Delta, Shandong province is a representative state to examine entrepreneurial activities; in fact, it has been investigated in previous related empirical studies (Yue and Mao, 2011; Kong et al., 2019; Chen et al., 2020). The entrepreneurial development in Shandong province has also been grounded in “local state entrepreneurship.” This development exhibits profit-seeking, readiness to take risks, sensitivity to market change, and high levels of rationality and efficiency, whereby entrepreneurs’ Darwinian social identity and business performance may drive entrepreneurial activities (Zhang, 1996).

A list of sample firms was acquired from the Human Resources and Social Security Department of Shandong province. With the support of the Chinese Academy of Labor and Social Security, a trained investigative team was tasked for data collection. An online survey was used in this study because of the COVID-19 pandemic. Of the 1,846 firms selected for the sample, 494 founders, co-founders, borders, or general managers participated in the online survey. Of those, 173 completed questionnaires were retained for data analysis, including only participants who are founders or co-founders of their firms, also named as entrepreneurs, to test the role of entrepreneurs’ Darwinian social identity on business performance. Table 1 shows the profile statistics of the 173 respondents in terms of their gender, age, and education, including their firm’s age, assets, and the number of employees.

**TABLE 1:** Sample description.

	Count	%	Cumulative %
**Gender**			
Male	122	29.480%	29.480%
Female	51	70.520%	100%
**Age**			
20–30	10	5.780%	5.780%
31–40	97	56.069%	61.850%
41–50	45	26.012%	87.861%
51–60	18	10.405%	98.266%
61 and above	3	1.734%	100%
**Education**			
Junior high school	10	5.780%	5.780%
High school or equal	42	24.277%	30.058%
College or bachelor’s degree	113	65.318%	95.376%
Postgraduate and above	8	4.624%	100%
**Firm age**			
0–1	8	4.624%	4.624%
2–5	110	63.584%	68.208%
6–10	35	20.231%	88.439%
11–15	11	6.358%	94.798%
16 and above	9	5.202%	100%
**Firm asset (10 thousands Chinese Yuan)**			
0–10	32	18.497%	18.497%
11–100	42	24.277%	42.775%
101–200	43	24.855%	67.630%
201–300	35	20.231%	87.861%
301 and above	20	11.561%	99.422%
Missing	1	0.578%	100%
**Number of employees**			
0–3	42	24.277%	24.277%
4–5	34	19.653%	43.931%
6–10	45	26.012%	69.942%
11–100	41	23.699%	93.642%
101 and above	11	6.358%	100%

Considering that the questionnaires of this study were collected from a single respondent, the common method bias might be a concern for this study. Common method bias was examined by conducting Harman’s single-factor test (Podsakoff et al., 2003; Wang et al., 2019, 2021). The un-rotated factor analysis shows that the first factor accounts for 42.678% of the variance, which is lower than the 50% cut-off. The test indicates that CMV is not a serious concern in this study.

### Measures

#### Dependent and Independent Variables

As a dependent variable in this study business performance (BP) was measured by five items adapted from Li and Atuahene-gima (2001), using a 7-point Likert scale (1 indicating “much worse” and 7 indicating “much better”). In line with previous research (Li and Atuahene-gima, 2001; Flynn et al., 2010; Tsai and Yang, 2013; Zhang et al., 2016), this study used subjective perceptual financial performance for its emphasis on entrepreneurs’ perceptions and evaluations. As an antecedent in the structural model, entrepreneurs’ Darwinian social identity (EDSI), which is the most generic type of entrepreneurs’ social identity with self-interested feature when engaging with others during the entrepreneurship, was measured by five items adapted from Sieger et al. (2016). Respondents rate the three main identity dimensions (their basic social motivation why they engage in entrepreneurship, their basis for self-evaluation how they judge themselves on, and their frame of reference where the self-worth is derived) adopted by Brewer and Gardner (1996), using a 7-point Likert scale (1 indicating “totally disagree” and 7 indicating “totally agree”) to capture the entrepreneurs’ Darwinian social identity.

#### Mediating and Moderating Variables

As the mediator, CSR was measured using two aspects adapted from Oberseder et al. (2014), that is, an internal and an external aspect. The former encompasses working conditions, non-discrimination of employees, and adequate remuneration, whereas the latter stresses the responsibility of economic contribution, job creation, local community assistance, and donation. A 7-point Likert scale was adopted with 11 items (1 indicating “totally disagree” and 7 indicating “totally agree”). Entrepreneurs’ well-being (EWB) is included in the hypothesis testing as a moderator and was measured with engagement and satisfaction aspects according to the scales of Zheng et al. (2015). Engagement is defined as a positive work-related state of mind, which is the opposite of burnout, and is often characterized by being strongly involved and happily engrossed in an entrepreneur’s work. By contrast, satisfaction refers to a cognitive and judgmental assessment of an entrepreneur’s work (Schaufeli et al., 2006; Del Libano et al., 2010). With the above two aspects, Entrepreneurs’ well-being is measured using a 7-point Likert scale with eight items (1 indicating “totally disagree” and 7 indicating “totally agree”).

##### Control Variables

Three individual-level variables and three firm-level variables were included as control variables presented in Table 1 according to Chen et al. (2020). Gender, age, and education were integrated into the analysis of the present study and are similar to those identified by Eggers and Lin (2015) and Chen et al. (2020). Firm age was measured by the logarithm of the difference between 2021 and the firm’s founding year because firm age is a natural count, and the log serves to mitigate outliers. Firm asset was measured by the logarithm of the firm’s total asset value.

## Results

### Correlation Matrix

Table 1 shows the descriptive statistics for the demographics of the sample, and Table 2 presents the correlations and descriptive statistics for the variables. The results of correlations among the variables indicate that all correlations are below 0.70. To assess the possibility of multicollinearity, the variance inflation factors (VIF) for the regression models of the indirect effects were calculated. The results show that the maximum VIF values in each regression model are far lower than 2, indicating that no serious concerns of multicollinearity problems exist.

**TABLE 2:** The results of correlation matrix and descriptive statistics.

	Mean	Std. Deviation	BP	CSR	EDSI	EWB	Gender	Age	Education	Firm Age	Firm Asset	Number of employees
BP	4.518	1.115	0.935									
CSR	6.138	0.800	0.311**	0.785								
EDSI	6.049	0.830	0.229**	0.585**	0.718							
EWB	5.699	0.980	0.431**	0.553**	0.493**	0.785						
Gender	0.295	0.457	−0.057	−0.135	−0.121	−0.135	–					
Age	2.462	0.825	0.010	−0.044	−0.116	−0.068	0.130	–				
Education	2.688	0.652	0.019	−0.019	−0.086	−0.053	0.076	−0.098	–			
Firm Age	0.640	0.327	0.099	0.160*	−0.025	0.089	0.055	0.401**	−0.070	–		
Firm Asset	1.949	0.719	0.322**	0.121	0.057	0.160*	−0.080	−0.007	0.132	0.042	–	
Number of employees	1.017	0.593	0.318**	0.123	0.080	0.144	−0.123	0.235**	0.118	0.258**	0.310**	–

n = 173. The square roots of AVEs are along the diagonal. Significance levels are two tailed with **p < 0.01; *p < 0.05. Correlations with absolute values greater than 0.02 are statistically significant at p < 0.01. Correlations listed below 0.02 are either statistically significant at p < 0.05 or not statistically significant. BP = business performance; CSR = corporate social responsibility; EDSI = entrepreneurs’ Darwinian social identity; EWB = entrepreneurs’ well-being.

### Reliability and Validity

To acquire a validated measure, the questionnaires in this study were all based on previously used scales in the literature (Wang et al., 2019). All the questionnaires were developed in English. Then, the English-version questionnaires were translated into Chinese initially by a scholar to collect the data in the Chinese context. Afterward, the Chinese-version questionnaires were back-translated into English and strictly checked to ensure conceptual equivalence (Brislin, 1970). Thereafter, a pilot study was conducted with 12 business heads to enhance measurement validity. After the pre-test of the preliminary version of the questionnaires, the questionnaires were revised in accordance to feedback.

To test the reliability of all the latent constructs, Cronbach’s α and composite reliability (CR) were computed. As shown in Table 3, the values of Cronbach’s α are 0.972, 0.831, 0.948, and 0.919 for business performance, entrepreneurs’ Darwinian social identity, corporate social responsibility, and entrepreneurs’ well-being, respectively, which are larger than the 0.700 cut-off value. The values of CR are 0.972, 0.840, 0.945, and 0.919, respectively, which are larger than the 0.700 threshold value. To test the convergent validity, the average variance extracted (AVE) was computed. As shown in Table 3, the values of AVE are 0.874, 0.515, 0.616, and 0.616 for business performance, entrepreneurs’ Darwinian social identity, corporate social responsibility, and entrepreneurs’ well-being, respectively, which are larger than the 0.500 cut-off value. To assess the discriminant validity, the squared roots of the AVE for all latent constructs were computed to be compared with all the corresponding correlations. Table 3 shows that all the squared roots of the AVE are greater than all the corresponding correlations, indicating that these measures have acceptable discriminant validity.

**TABLE 3:** Measures.

Constructs and items	Loading
**Business performance (α = 0.972, CR = 0.972, AVE = 0.874)**	
Return on investment	0.961
Return on sales	0.953
Return on assets	0.954
Profit growth	0.917
Overall income	0.888
**Entrepreneurs’ Darwinian social identity (α = 0.831, CR = 0.840, AVE = 0.515)**	
I create my firm in order to advance my career in the business world	0.586
As a firm founder, it is very important for me to operate my firm on the basis of solid management practices	0.708
As a firm founder, it is very important for me to have thoroughly analyzed the financial prospects of my business	0.719
When managing my firm, it is very important for me to have a strong focus on what my firm can achieve vis-à-vis the competition	0.851
When managing my firm, it is very important for me to establish a strong competitive advantage and significantly outperform other firms in my domain	0.701
**Corporate social responsibility (α = 0.948, CR = 0.945, AVE = 0.616)**	
Set decent working conditions	0.783
Treat employees equally	0.907
Offer adequate remuneration	0.912
Develop, support and train employees	0.934
Communicate openly and honestly with employees	0.931
Contribute to the economic development of the region	0.605
Create jobs for people in the region	0.718
Respect regional values, customs, and culture	0.723
Make donations to social facilities	0.586
Support employees who are involved in social projects during working hours	0.714
Contribute to solving societal problems	0.715
**Entrepreneurs’ well-being (α = 0.919, CR = 0.919, AVE = 0.595)**	
I am enthusiastic about my job	0.530
I am immersed in my work	0.568
I get carried away when I am working	0.641
In most ways my work is close to my ideal	0.837
The conditions of my work are excellent	0.863
I am satisfied with my work	0.920
So far I have gotten the important things I want in my work	0.916
If I could change my work, I would change almost nothing	0.783

### Hypothesis Testing

In this study, the total effect (H1) and the indirect effect through CSR (H2a, H2b) are tested using the hierarchical regression analysis in SPSS 25. In addition, the mediating effect is tested using PROCESS model 4, which was used with 5000 bootstraps at the 95% confidence level. The moderating effects (H3a and H3b) are then tested using PROCESS v3.3 embedded in SPSS 25. PROCESS model 58 was utilized to test the moderating effects of entrepreneurs’ well-being on the relationship between entrepreneurs’ Darwinian social identity and CSR, as well as the relationship between CSR and business performance with a 95% confidence level. Results are shown in Tables 4, 5.

**TABLE 4:** The direct and indirect effects of entrepreneurs’ Darwinian social identity.

	Business performance			Corporate social responsibility	
	Model 1	Model 2	Model 3	Model 4	Model 5
Constant	3.637***	1.924*	1.219	6.006***	2.342***
EDSI		0.260**	0.093		0.555***
CSR			0.301*		
Gender	0.010	0.046	0.082	−0.197	−0.119
Age	−0.098	−0.061	−0.048	−0.121	−0.041
Education	−0.081	−0.045	−0.058	−0.034	0.044
Firm Age	0.168	0.168	0.025	0.476*	0.477**
Firm Asset	0.385**	0.376**	0.353**	0.096	0.077
Number of employees	0.473**	0.434**	0.433**	0.087	0.003
*R* ^2^	0.163	0.199	0.227	0.071	0.387
*△R^2^*	0.163	0.036	0.029	0.071	0.317
*F-Value*	5.385***	5.838***	6.021***	2.106^†^	14.908***
*Max VIF*	1.268	1.286	1.632	1.268	1.286

^n = 173. The significance is two tailed with †^p < 0.100; *p < 0.050; **p < 0.010; ***p < 0.001.

**TABLE 5:** The mediating effect test of corporate social responsibility.

Mediators	Effect	BootSE	[BootLLCI, BootULCI]
**The mediating effect between entrepreneurs’ Darwinian social identity and** **business performance**			
Indirect effect	0.167	0.070	[0.042, 0.312]

*n = 173. CI_95%_, confidence interval with confident level as 95%. Based on PROCESS model 4.*

As shown in Table 4, the effect of entrepreneurs’ Darwinian social identity on business performance is positive and significant (Model 2: β = 0.260, *p* < 0.010), thereby supporting H1. In addition, entrepreneurs’ Darwinian social identity has a positive and significant impact on CSR (Model 5: β = 0.555, *p* < 0.001), and CSR is positively and significantly related to business performance, with entrepreneurs’ Darwinian social identity being controlled (Model 3: β = 0.301, *p* < 0.050). Therefore, in H2a and H2b, the mediating effect of CSR in the relationship between entrepreneurs’ Darwinian social identity and business performance, is supported. Table 5 shows that the indirect effects of entrepreneurs’ Darwinian social identity and business performance through CSR are also significant (indirect effect = 0.167, 95% CI = [0.042, 0.312]). Thus, these results support the mediating effect of CSR.

Regarding the moderating effects, entrepreneurs’ Darwinian social identity, CSR, and entrepreneurs’ well-being are mean-centered before entering into regression for better interpretations. The results in Table 6 indicate that entrepreneurs’ well-being negatively and significantly moderates the effect of entrepreneurs’ Darwinian social identity on CSR (Model 6: β = –0.170, 95% CI = [–0.285, –0.055]). By contrast, the moderating effect of entrepreneurs’ well-being is positive and significant as for the relationship between CSR and business performance (Model 7: β = 0.216, 95% CI = [0.012, 0.421]). Thus, H3a and H3b are supported.

**TABLE 6:** The moderating effect of entrepreneurs’ well-being.

	Corporate social responsibility			Business performance		
	Model 6			Model 7		
	Coeff. (SE)	*p* value	95%CI	Coeff. (SE)	*p* value	95%CI
Constant	−0.275(0.270)	0.310	[−0.809, 0.259]	3.566(0.438)	0.000	[2.701, 4.431]
EDSI	0.370(0.064)	0.000	[0.242, 0.497]	0.005(0.114)	0.964	[−0.220, 0.231]
CSR				0.232(0.133)	0.084	[−0.031, 0.494]
EWB	0.285(0.055)	0.000	[0.177, 0.393]	0.317(0.097)	0.001	[0.126, 0.509]
EDSI × EWB	−0.170(0.058)	0.004	[−0.285, −0.055]			
CSR × EWB				0.216(0.103)	0.038	[0.012, 0.421]
Gender	−0.099(0.102)	0.333	[−0.299, 0.102]	0.113(0.165)	0.492	[−0.212, 0.439]
Age	−0.014(0.062)	0.827	[−0.136, 0.109]	−0.030(0.100)	0.765	[−0.228, 0.168]
Education	0.056(0.071)	0.432	[−0.085, 0.197]	−0.018(0.116)	0.877	[−0.247, 0.211]
Firm Age	0.368(0.154)	0.018	[0.063, 0.672]	0.021(0.254)	0.935	[−0.481, 0.523]
Firm Asset	0.022(0.067)	0.741	[−0.110, 0.154]	0.270(0.110)	0.015	[0.054, 0.487]
Number of employees	−0.024(0.086)	0.784	[−0.193, 0.146]	0.401(0.139)	0.004	[0.127, 0.675]
	R^2^ = 0.488, F(9, 163) = 17.237, *p* = 0.000			R^2^ = 0.314, F(10, 162) = 7.418, *p* = 0.000		

*n = 173. 95% CI, confident interval with confidence level of 95%. The results were obtained based on PROCESS model 58.*

### Supplemental Analysis

**FIGURE 2:**
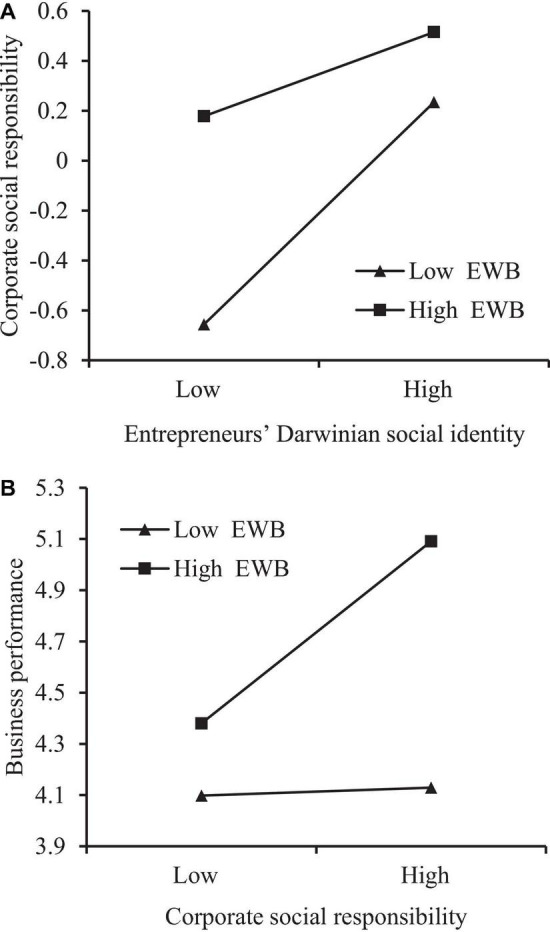
**(A)** The negative moderating effect of entrepreneurs’ well-being. **(B)** The positive moderating effect of entrepreneurs’ well-being.

To probe the moderating effect of entrepreneurs’ well-being, the pick-a-point approach was used (Figures 2a,b). In Figure 2a, the positive effect of entrepreneurs’ Darwinian social identity on CSR appears to weaken when entrepreneurs’ well-being is high, which is consistent with H3a. In Figure 2b, the positive effect of CSR on business performance appears to strengthen when entrepreneurs’ well-being is high, which is consistent with H3b.

## Discussion and Conclusion

### Entrepreneurs’ Darwinian Social Identity and Its Impact on Business Performance

An increasing number of studies have examined the relationship between entrepreneurs’ social identity and business performance (Fauchart and Gruber, 2011; de la Cruz et al., 2018; Wagenschwanz, 2021). However, little is still known with regard to the influence of entrepreneurs’ Darwinian social identity on business performance. As such, this study examines the relationship between entrepreneurs’ Darwinian social identity on business performance through CSR contingent on the effect of entrepreneurs’ well-being grounding in social identity theory and stakeholder theory.

Previous studies of entrepreneurs’ Darwinian social identity indicated that entrepreneurs with Darwinian social identity regard competitors as their reference to evaluate themselves (Fauchart and Gruber, 2011). From such a perspective, the goal of entrepreneurs is to pursue success by achieving superior business performance (de la Cruz et al., 2018). However, business success cannot be only achieved through financial performance improvement directly, but also through the social and non-financial aspects indirectly, such as employee satisfaction and broader contributions to the community and society as a whole, which is broadly categorized as CSR. As such both direct and indirect effect of entrepreneurs’ Darwinian social identity on business performance are investigated in the present study.

With data collected from the Shandong province of China through a larger-scale questionnaire survey, this study found that the empirical results support the hypotheses. The findings demonstrate that entrepreneurs’ Darwinian social identity has a positive effect on business performance. Furthermore, with respect to the role of entrepreneurial identity in the acquisition of a firm’s social and non-financial success, the findings indicate that entrepreneurs’ Darwinian social identity positively impacts CSR, which in turn, positively contributes to business performance. In addition, to disclose the effect of entrepreneurs’ well-being on business performance because of the entrepreneurs’ Darwinian social identity, the relationship between entrepreneurs’ Darwinian social identity and CSR is negatively moderated, whereas the relationship between CSR and business performance is positively moderated by entrepreneurs’ well-being.

### Contributions

This study contributes to literatures with regard to entrepreneurs’ identity in several ways. First, this study introduce the non-financial mechanism to enrich the theoretical logic how entrepreneurs’ Darwinian social identity affects business performance. Previous studies on the entrepreneurs’ identity indicate that the entrepreneurs with Darwinian social identity regard competitors as their reference to evaluate themselves. As such, the goal of entrepreneurs is to pursue success by acquire superior financial performance directly (Fauchart and Gruber, 2011; de la Cruz et al., 2018). In fact, the mechanism of entrepreneurs’ social identity on business performance involve not only the direct aspects to business performance, such as financial performance and entrepreneurship failure, but also the indirect aspects, such as CSR and growth (Wiklund et al., 2019; Amorós et al., 2021). However, only a few studies examined the indirect channel between entrepreneurs’ Darwinian social identity and business performance. In addition, in business-society relations of China, CSR is considered doubly oxymoronic because of the complex nature of the business and social environment, along with the fast-changing cultural and political progress (Moon and Shen, 2010). In the Chinese context, a significant research question exists between the importance of CSR in fostering healthy and sustainable business-society relations and social license to operate and the lack of understanding of implicit and explicit motives that drive CSR measures including how CSR relates to business performance (Moon and Shen, 2010; Yu et al., 2021). Thus, the results indicate that CSR is a significant channel that entrepreneurs’ Darwinian social identity influences business performance, which enriches the theoretical logic underlying the relationship between entrepreneurs’ Darwinian social identity and business performance.

Second, this present study enriches social identity theory by adding a broaden-and-build perspective to investigate the role of entrepreneurs’ well-being. With the increasing attention paid by scholars, entrepreneurs’ well-being as a critical psychological resource has a significant impact on the functioning of entrepreneurs’ social identity (Wiklund et al., 2019). However, few studies have probed the role of entrepreneurs’ well-being on the functioning of entrepreneurs’ social identity. The present study bridges this gap by adding a broaden-and-build perspective into social identity theory (Fredrickson, 2001). On the relationship between entrepreneurs’ Darwinian social identity and CSR, a broaden-and-build perspective was added into the social identity theory by arguing that entrepreneurs with well-being pursue long-term benefits including CSR with more freedom (Cardon et al., 2009; Baron et al., 2016). This perspective, in turn, makes entrepreneurs more focused on “meaningful work” to support employees’ growth and social development. In addition, in the relationship between CSR and business performance, according to a broaden-and-build perspective added into the social identity theory, three mechanisms that include flexible and adaptive components in adverse situations (Ryff, 2019), high self-efficacy (Kolvereid and Bullvag, 1996; Bradley and Roberts, 2004), and engagement and commitment (Benz and Frey, 2008; Felfe and Heinitz, 2008), were used to argue the positive moderating effect of entrepreneurs’ well-being. The results indicate both negative and positive moderating effects of entrepreneurs’ well-being on the relationship between entrepreneurs’ identity and business performance through CSR. Thus, our understanding of the underlying mechanism is enhanced by enriching social identity theory with a broaden-and-build perspective.

Third, this study adds to theory integration theory by combining social identity theory and stakeholder theory to enhance the ability of the theory to reflect the reality of management (Suddaby et al., 2011; Mayer and Sparrowe, 2013). Literatures regarding theory integration indicate that two or more theories can be combined together according to their underlying logic (Suddaby et al., 2011; Mayer and Sparrowe, 2013). With the dimensions underlying the relationship between theories such as proximity and compatibility, four different types of theory integration have been examined in previous studies (Suddaby et al., 2011; Mayer and Sparrowe, 2013). However, there is still a dearth of research to probe how to integrate three theories together to explain the reality of management. This study analyzes the underlying mechanism of entrepreneurs’ Darwinian social identity and its impact on business performance. In particular, entrepreneurs’ Darwinian social identity makes them exceed competitors and other entrepreneurs with successful businesses with the underlying logic of social identity theory. By contrast, firms prefer long-term sustainable and social responsibility issues rather than short-term financial profits to share more value with stakeholders according to stakeholder theory. By combing these two theories, an entrepreneur with a Darwinian social identity intends to pursue a successful business by extending the entrepreneur’s self into society, which, in turn, influences the success of entrepreneurship. The results of this study indicate a combining mode of theory integration to discourse the underlying mechanism between entrepreneurs’ Darwinian social identity and business performance via through CSR and thus contribute to the literature of theory integration.

Fourth, the impact of entrepreneurs’ Darwinian social identity on CSR suggests entrepreneurs’ characteristics, personalities, and identities playing important roles of shaping corporate citizenship. This sheds lights on centering entrepreneurs in understanding the development of corporate citizenship, while existing literature on corporate citizenship theory has largely considered corporate citizenship as an extension of the corporatism (Jones and Haigh, 2007), rather than an extension of its founders’ selves. Therefore, this study provides an angle to link social identity theory and corporate citizenship theory, in discussing CSR matters.

In addition to its theoretical contributions, this study has critical implications for practice. First, the social and non-financial success impact, such as CSR, induced by entrepreneurs’ Darwinian social identity provides entrepreneurs with a new channel to achieve business success. Regardless of an entrepreneur’s goal to establish a business entity, non-financial success such as CSR can provide meaning to those seeking to fulfill their Darwinian social identity. CSR has become significant influencing firms and organizations’ reputations in the society, and further their abilities of surviving and thriving in contemporary business world. The consequence is that firms care more about maximizing their profits and may sacrifice their claimed social responsibilities. Considering the circumstance where stress for material pursuits dominate, it is therefore critical to develop an in-depth understand on the role of CSR in business development in contexts like China, and how CSR and business performance relate to each other. The results in this study may prove beneficial to encouraging the adoption of CSR in China where the concept has previously received only limited attention in business practices, so that entrepreneurs can acquire business success via indirect channel of CSR.

Second, the role of entrepreneurs’ Darwinian well-being as a psychological resource inspires the entrepreneurs to care about the effect of their well-being, when pursuing business performance based on their Darwinian social identity via indirect channel of CSR. Entrepreneurs’ well-being has been regarded as a key outcome in entrepreneurship for entrepreneurs to take part in entrepreneurial activities. As a matter of fact, entrepreneurs’ well-being is not only a critical outcome variable, but also an important antecedent variable. As an antecedent variable, the effect of entrepreneurs’ Darwinian well-being incudes the direct role on entrepreneurs themselves and the indirect spillover role on stakeholders. In this study, both direct and indirect roles were investigated according to the logic of theory integration. Interestingly, on the one hand, entrepreneurs’ well-being could impede the beneficial effect of entrepreneurs’ Darwinian social identity such as weakening the impact on CSR; on the other hand, entrepreneurs’ well-being could enhance the transformation from a non-profitable success to profitable success of entrepreneurs’ Darwinian social identity. As such, entrepreneurs should balance the positive effect and negative effect on business success when caring about their well-being, in order to use their “freedom” to achieve the goal of their well-being.

### Limitations and Future Research

Despite the abovementioned contribution, this study has several limitations that open new research opportunities. The first limitation is the sample. Cross-sectional rather than longitudinal data were used to test causal inferences in this study. Future studies could collect longitudinal data so that the causal inferences could be more reliably investigated. The second limitation is the measurement. Individual- and firm-level variables were included and measured with the same questionnaire in the theoretical concept model according to upper echelons theory. Future research designs could combine individual-level measurement from entrepreneurs with the measurement at the firm level. In addition, hierarchical multiple regression rather than ordinary multiple regression could be used. Future studies could also examine entrepreneurs’ communitarian and missionary identities to broaden the understanding of entrepreneurial identity.

## Data Availability Statement

The raw data supporting the conclusions of this article will be made available by the authors, without undue reservation.

## Ethics Statement

The studies involving human participants were reviewed and approved by the Academic Committee of School of Agricultural Economics and Rural Development in Renmin University of China. Written informed consent for participation was not required for this study in accordance with the national legislation and the institutional requirements.

## Author Contributions

JC participated in design, data collection, drafting of the first version, and revision of the article. NC participated in design, drafting of the first version, and revision of the article. KY participated in design, data collection, drafting of the first version, and revision of the article. CMH participated in revision of the article and important intellectual input. All authors contributed to the article and approved the submitted version.

## Conflict of Interest

The authors declare that the research was conducted in the absence of any commercial or financial relationships that could be construed as a potential conflict of interest.

## Publisher’s Note

All claims expressed in this article are solely those of the authors and do not necessarily represent those of their affiliated organizations, or those of the publisher, the editors and the reviewers. Any product that may be evaluated in this article, or claim that may be made by its manufacturer, is not guaranteed or endorsed by the publisher.

## References

[B1] Allouche J. Laroche P. (2005). A meta-analytical investigation of the relationship between corporate social and financial performance. *Rev. Gestion Ressour. Hum.* 57 18–40.

[B2] Alsos G. A. Clausen T. H. Hytti U. Solvoll S. (2016). Entrepreneurs’ social identity and the preference of causal and effectual behaviours in start-up processes. *Entrep. Reg. Dev.* 28 234–258.

[B3] Amorós J. E. Cristi O. Naudé W. (2021). Entrepreneurship and subjective well-being: does the motivation to start-up a firm matter? *J. Bus. Res.* 127 389–398.

[B4] Ashforth B. E. Mael F. (1989). Social identity theory and the organization. *Acad. Manag. Rev.* 14 20–39. 10.1017/cbo9781139136983.004

[B5] Barnett M. L. (2007). Stakeholder influence capacity and the variability of financial returns to corporate social responsibility. *Acad. Manag. Rev.* 32 794–816. 10.5465/amr.2007.25275520

[B6] Baron R. A. Franklin R. J. Hmieleski K. M. (2016). Why entrepreneurs often experience low, not high, levels of stress: the joint effects of selection and psychological capital. *J. Manag.* 42 742–768. 10.1177/0149206313495411

[B7] Benz M. Frey B. S. (2008). Being independent is a great thing: subjective evaluations of self-employment and hierarchy. *Economica* 75 362–383. 10.1111/j.1468-0335.2007.00594.x

[B8] Bolourian S. Angus A. Alinaghian L. (2021). The impact of corporate governance on corporate social responsibility at the board-level: a critical assessment. *J. Clean. Prod.* 291:125752.

[B9] Bradley D. E. Roberts J. A. (2004). Self-employment and job satisfaction: investigating the role of self-efficacy, depression, and seniority. *J. Small Bus. Manag.* 42 37–58. 10.1111/j.1540-627x.2004.00096.x

[B10] Brewer M. B. Gardner W. (1996). Who is this “we”? Levels of collective identity and self- representations. *J. Pers. Soc. Psychol.* 71 83–93. 10.1037/0022-3514.71.1.83

[B11] Brislin R. W. (1970). Back-translation for cross-cultural research. *J. Cross Cult. Psychol.* 1 185–216. 10.1037/a0021453 21038953

[B12] Bulmash B. (2016). Entrepreneurial resilience: locus of control and well-being of entrepreneurs. *J. Entrep. Organ. Manag.* 05 1–6.

[B13] Burke L. Logsdon J. M. (1996). How corporate social responsibility pays off. *Long Range Plann.* 29 495–502. 10.1016/0024-6301(96)00041-6

[B14] Cahan S. Chen C. Chen L. Nguyen N. H. (2015). Corporate social responsibility and media coverage. *J. Bank. Financ.* 59 19–27.

[B15] Cardon M. S. Patel P. C. (2015). Is stress worth it? Stress-related health and wealth trade-offs for entrepreneurs. *Appl. Psychol.* 64 379–420. 10.1111/apps.12021

[B16] Cardon M. S. Wincent J. Singh J. Drnovsek M. (2009). The nature and experience of entrepreneurial passion. *Acad. Manag. Rev.* 34 511–532. 10.5465/amr.2009.40633190

[B17] Carroll A. (1979). Three dimensional conceptual model of corporate performance. *Acad. Manag. Rev.* 4 497–505. 10.2307/257850

[B18] Caudill E. (2005). *Darwinian Myths: The Legends and Misuses of a Theory.* Knoxville, TN: University of Tennessee Press.

[B19] Chen J. Jiang F. Lin S. (2020). How coping combination affects innovation ambidexterity in business failure situations. *Front. Psychol.* 11:1409. 10.3389/fpsyg.2020.01409 32765339 PMC7381210

[B20] Chen N. C. Hall C. M. Prayag G. (2021). *Sense of Place and Place Attachment in Tourism.* Milton Park: Routledge.

[B21] Cinar E. M. Du Y. Hienkel T. (2018). Chinese entrepreneurship attributes: a comparative GEM data analysis. *J. Entrep. Emerg. Econ.* 10 217–248. 10.1108/jeee-03-2017-0016

[B22] de la Cruz M. E. Jover A. J. V. Gras J. M. G. (2018). Influence of the entrepreneur’s social identity on business performance through effectuation. *Eur. Res. Manag. Bus. Econ.* 24 90–96. 10.1016/j.iedeen.2017.11.003

[B23] Del Libano M. Llorens S. Salanova M. Schaufeli W. B. (2010). Validity of a brief workaholism scale. *Psicothema* 22 143–150. 20100441

[B24] Deng W. Liang Q. Z. Fan P. H. (2019). Complements or substitutes? Configurational effects of entrepreneurial activities and institutional frameworks on social well-being. *J. Bus. Res.* 96 194–205. 10.1016/j.jbusres.2018.11.003

[B25] Eggers J. P. Lin S. (2015). Dealing with failure: serial entrepreneurs and the costs of changing industries between ventures. *Acad. Manag. J.* 58 1785–1803.

[B26] Fauchart E. Gruber M. (2011). Darwinians, communitarians, and missionaries: the role of founder identity in entrepreneurship. *Acad. Manag. J.* 54 935–957.

[B27] Felfe J. Heinitz K. (2008). “The impact of followers’ and leaders’ personality and perceived simi- larity on followers’ perceptions of transformational leadership and leader acceptance,” in *Personality and Work München*, ed. Deller J. (Mering: Rainer Hampp Verlag), 197–217.

[B28] Flynn B. B. Huo B. Zhao X. (2010). The impact of supply chain integration on performance: a contingency and configuration approach. *J. Oper. Manag.* 28 58–71. 10.1016/j.jom.2009.06.001

[B29] Fredrickson B. L. (2001). The role of positive emotions in positive psychology: the broaden and-build theory of positive emotions. *Am. Psychol.* 56 218–226. 11315248 10.1037//0003-066x.56.3.218 PMC3122271

[B30] Freudenreich B. Lüdeke-Freund F. Schaltegger S. (2020). A stakeholder theory perspective on business models: value creation for sustainability. *J. Bus. Ethics* 166 3–18. 10.1007/s10551-019-04112-z

[B31] Galbreath J. Shum P. (2012). Do customer satisfaction and reputation mediate the CSR–FP link? Evidence from Australia. *Aust. J. Manag.* 37 211–229. 10.1177/0312896211432941

[B32] Gillan S. L. Koch A. Starks L. T. (2021). Firms and social responsibility: a review of ESG and CSR research in corporate finance. *J. Corp. Financ.* 66:101889.

[B33] Grimes M. G. (2018). The pivot: how founders respond to feedback through idea and identity work. *Acad. Manag. J.* 61 1692–1717. 10.5465/amj.2015.0823

[B34] Hahn V. C. Frese M. Binnewies C. Schmitt A. (2012). Happy and proactive? The role of hedonic and eudaimonic well-being in business owners’ personal initiative. *Entrep. Theory Pract.* 36 97–114.

[B35] Hall C. M. Williams A. M. (2020). *Tourism and Innovation*, 2nd Edn. London: Routledge.

[B36] Hambrick D. C. Mason P. A. (1984). Upper echelons: the organization as a reflection of its top managers. *Acad. Manag. Rev.* 9 193–206. 10.5465/amr.1984.4277628

[B37] Hobfoll S. E. (2001). The influence of culture, community, and the nested-self in the stress process: advancing conservation of resources theory. *Appl. Psychol.* 50 337–421.

[B38] Hoggy M. Terry D. J. (2000). Social identity and self-categorization processes in organizational context. *Acad. Manag. Rev.* 25 121–140.

[B39] Johnson H. H. (2003). Does it pay to be good? Social responsibility and financial performance. *Bus. Horiz.* 46 34–40. 10.1016/s0007-6813(03)00086-7

[B40] Jones M. T. Haigh M. (2007). The transnational corporation and new corporate citizenship theory: a critical analysis. *J. Corp. Citizensh.* 27 51–69. 10.9774/gleaf.4700.2007.au.00007

[B41] Jones T. M. (1995). Instrumental stakeholder theory: a synthesis of ethics and economics. *Acad. Manag. Rev.* 20 404–437. 10.5465/amr.1995.9507312924

[B42] Ko E. J. Kim K. (2020). Connecting founder social identity with social entrepreneurial intentions. *Soc. Enterp. J.* 16 403–429. 10.1108/sej-02-2020-0012

[B43] Kolvereid L. Bullvag E. (1996). Growth intentions and actual growth: the impact of entrepreneurial choice. *J. Enterp. Cult.* 4 1–17. 10.1142/s0218495896000022

[B44] Kong F. (2017). On influences of self-identification on entrepreneurial motivation: self-efficacy as the moderator. *J. Changzhou Univ. Soc. Sci. Ed.* 10 50–58.

[B45] Kong F. Z. Zhao L. Zhang X. B. Tsai C. H. Lin D. D. (2019). Farmers’ work-life quality and entrepreneurship will in China. *Front. Psychol.* 10:787. 10.3389/fpsyg.2019.00787 31114517 PMC6502897

[B46] Lewis P. (2013). The search for an authentic entrepreneurial identity: difference and professionalism among women business owners. *Gend. Work Organ.* 20 252–266. 10.1111/j.1468-0432.2011.00568.x

[B47] Li H. Atuahene-gima K. (2001). Product innovation strategy and the performance of new technology ventures in China. *Acad. Manag. J.* 44 1123–1134. 10.2307/3069392

[B48] Malik M. (2015). Value-enhancing capabilities of CSR: a brief review of contemporary literature. *J. Bus. Ethics* 127 419–438. 10.1007/s10551-014-2051-9

[B49] Margolis J. D. Walsh J. P. (2003). Misery loves companies: rethinking social initiatives by business. *Adm. Sci. Q.* 48 268–305.

[B50] Mayer K. J. Sparrowe R. T. (2013). From the editors: integrating theories in AMJ articles. *Acad. Manag. J.* 56 917–922. 10.5465/amj.2013.4004

[B51] McGee J. E. Peterson M. Mueller S. L. Sequeira J. M. (2009). Entrepreneurial self-efficacy: refining the measure. *Entrep. Theory Pract.* 33 965–988.

[B52] Miller D. Breton-Miller I. L. (2011). Governance, social identity, and entrepreneurial orientation in closely held public companies. *Soc. Sci. Electron. Publ.* 35 1051–1076. 10.1111/j.1540-6520.2011.00447.x

[B53] Mio C. Costantini A. Panfilo S. Baggio S. (2020). CSR and management control integration. Evidence from an employee welfare plan implementation. *Manag. Control* 1 151–175.

[B54] Moon J. Shen X. (2010). CSR in China research: salience, focus and nature. *J. Bus. Ethics* 94 613–629.

[B55] Moser D. V. Martin P. R. (2012). A broader perspective on corporate social responsibility research in accounting. *Account. Rev.* 87 797–806. 10.2308/accr-10257

[B56] Murnieks C. Mosakowski E. (2007). Who am: I? Looking inside the “entrepreneurial identity”. *Front. Entrep. Res.* 27:1–14. 10.4337/9781785363719.00007

[B57] Murnieks C. Y. Mosakowski E. Cardon M. S. (2014). Pathways of passion: identity centrality, passion, and behavior among entrepreneurs. *J. Manag.* 40:0149206311433855.

[B58] Ni V. Davidson H. (2021). *China’s cultural Crackdown: Few Areas Untouched as Xi Reshapes Society. The Guardian.* Available online at: https://www.theguardian.com/world/2021/sep/10/chinas-cultural-crackdown-few-areas-untouched-as-xi-reshapes-society (accessed September 10, 2021)

[B59] Nielsen K. Yarker J. Randall R. Munir F. (2009). The mediating effects of team and self-efficacy on the relationship between transformational leadership, and job satisfaction and psychological well-being in healthcare professionals: a cross-sectional questionnaire survey. *Int. J. Nurs. Stud.* 46 1236–1244. 10.1016/j.ijnurstu.2009.03.001 19345946

[B60] Nikolaev B. Boudreaux C. J. Wood M. (2020). Entrepreneurship and subjective well-being: the mediating role of psychological functioning. *Entrep. Theory Pract.* 44 557–586. 10.1177/1042258719830314

[B61] Oberseder M. Schlegelmilch B. B. Murphy P. E. Gruber V. (2014). Consumers’ perceptions of corporate social responsibility: scale development and validation. *J. Bus. Ethics* 124 101–115. 10.1007/s10551-013-1787-y

[B62] Orlitzky M. Schmidt F. L. Rynes S. L. (2003). Corporate social and financial performance: a meta-analysis. *Organ. Stud.* 24 403–441.

[B63] Pan N. D. Gruber M. Binder J. (2019). Painting with all the colors: the value of social identity theory for understanding social entrepreneurship. *Acad. Manag. Rev.* 44 213–215. 10.5465/amr.2017.0504

[B64] Patzelt H. Shepherd D. A. (2011). Negative emotions of an entrepreneurial career: self-employment and regulatory coping behaviors. *J. Bus. Ventur.* 26 226–238.

[B65] Podsakoff P. M. MacKenzie S. B. Lee J. Y. Podsakoff N. P. (2003). Common method biases in behavioral research: a critical review of the literature and recommended remedies. *J. Appl. Psychol.* 88 879–903. 10.1037/0021-9010.88.5.879 14516251

[B66] Ponzi L. J. Fmbrun C. J. Gardberg N. A. (2011). RepTrak pulse: conceptualizing and validating a short form measure of corporate reputation. *Corp. Reput. Rev.* 14 15–35. 10.1057/crr.2011.5

[B67] Porter M. E. Kramer M. R. (2002). The competitive advantage of corporate philanthropy. *Harvard Bus. Rev.* 80 56–65. 12510538

[B68] Powell E. E. Baker T. (2014). It’s what you make of it: founder identity and enacting strategic responses to adversity. *Acad. Manag. J.* 57 1406–1433. 10.5465/amj.2012.0454

[B69] Preston L. E. O’Bannon D. P. (1997). The corporate social-financial performance relationship: a typology and analysis. *Bus. Soc.* 36 419–429.

[B70] Rindova V. Barry D. Ketchen D. J. (2009). Introduction to special topic forum: entrepreneuring as emancipation. *Acad. Manag. Rev.* 34 477–491. 10.5465/amr.2009.40632647

[B71] Rodriguez-Gomez S. Arco-Castro M. L. Lopez-Perez M. V. Rodríguez-Ariza L. (2020). Where does CSR come from and where does it go? A review of the state of the art. *Adm. Sci.* 10 1–19.

[B72] Ryan R. M. Deci E. L. (2001). On happiness and human potentials: a review of research on hedonic and eudaimonic well-being. *Annu. Rev. Psychol.* 52 141–166. 10.1146/annurev.psych.52.1.141 11148302

[B73] Ryff C. D. (2019). Entrepreneurship and eudaimonic well-being: five venues for new science. *J. Bus. Ventur.* 34 646–663. 10.1016/j.jbusvent.2018.09.003 31105380 PMC6516495

[B74] Saeidi S. P. Sofian S. Saeidi P. Saeidi S. P. Saaeidi S. A. (2015). How does corporate social responsibility contribute to firm financial performance? The mediating role of competitive advantage, reputation, and customer satisfaction. *J. Bus. Res.* 68 341–350. 10.1016/j.jbusres.2014.06.024

[B75] Schaufeli W. B. Bakker A. Salanova M. (2006). The measurement of work engagement with a short questionnaire: a cross-national study. *Educ. Psychol. Meas.* 66 701–716. 10.1177/0013164405282471

[B76] Schuler D. A. Cording M. (2006). A corporate social performance-corporate financial performance behavioral model for consumers. *Acad. Manag. Rev.* 31 540–558.

[B77] Seibert S. E. Nielsen J. D. Kraimer M. L. (2021). Awakening the entrepreneur within: entrepreneurial identity aspiration and the role of displacing work events. *J. Appl. Psychol.* 106 1224–1238. 10.1037/apl0000823 32881538

[B78] Shir N. Nikolaev B. N. Wincent J. (2019). Entrepreneurship and well-being: the role of psychological autonomy, competence, and relatedness. *J. Bus. Ventur.* 34:105875.

[B79] Sieger P. Gruber M. Fauchart E. Zellweger T. (2016). Measuring the social identity of entrepreneurs: scale development and international validation. *J. Bus. Vent.* 31 542–557. 10.1016/j.jbusvent.2016.07.001

[B80] Smith I. H. Woodworth W. P. (2012). Engaging the informal economy to educate social entrepreneurs and social innovators. *Acad. Manag. Proc.* 2012:14188. 10.1111/hex.12205 24826905 PMC5810666

[B81] Snider J. Hill R. P. Martin D. (2003). Corporate social responsibility in the 21st century: a view from the world’s most successful firms. *J. Bus. Ethics* 48 175–187.

[B82] Stajkovic A. D. Luthans F. (1998). Self-efficacy and work-related performance: a meta-analysis. *Psychol. Bull.* 124 240–261. 10.1037/0033-2909.124.2.240

[B83] Stephan U. (2018). Entrepreneurs’ mental health and well-being: a review and research agenda. *Acad. Manag. Perspect.* 32 290–322. 10.5465/amp.2017.0001

[B84] Su X. Xiao J. Chen J. (2020). Social identity recognition of entrepreneurs and innovation of new enterprises. *S. China J. Econ.* 39 108–124.

[B85] Suddaby R. Hardy C. Huy Q. N. (2011). Introduction to special topic forum—where are the new theories of organization? *Acad. Manag. Rev.* 36 236–246.

[B86] Swanson D. L. (1995). Addressing a theoretical problem by reorienting the corporate social performance model. *Acad. Manag. Rev.* 20 43–64. 10.2307/258886

[B87] Tsai K. H. Yang S. Y. (2013). Firm innovativeness and business performance: the joint moderating effects of market turbulence and competition. *Ind. Mark. Manag.* 42 1279–1294. 10.1016/j.indmarman.2013.06.001

[B88] Turker D. (2009). How corporate social responsibility influences organization commitment. *J. Bus. Ethics* 89 189–204. 10.1007/s10551-008-9993-8

[B89] Uy M. A. Foo M. D. Song Z. (2013). Joint effects of prior start-up experience and coping strategies on entrepreneurs’ psychological well-being. *J. Bus. Ventur.* 28 583–597. 10.1016/j.jbusvent.2012.04.003

[B90] Van Beurden P. T. Gossling B. T. (2008). The worth of values: a literature review on the relation between corporate social and financial performance. *J. Bus. Ethics* 82 407–424. 10.1007/s10551-008-9894-x

[B91] Van der Laan G. Van Ees H. Van Witteloostuijn A. (2008). Corporate social and financial performance: an extended stakeholder theory, and empirical test with accounting measures. *J. Bus. Ethics* 79 299–310. 10.1007/s10551-007-9398-0

[B92] Van Praag C. M. (1999). Some classic views on entrepreneurship. *Economist* 147 311–335.

[B93] Velte P. (2021). Meta-analyses on corporate social responsibility (CSR): a literature review. *Manag. Rev. Q.* 1–49. 10.1007/s11301-021-00211-2

[B94] Wagenschwanz A. M. (2021). The identity of entrepreneurs: providing conceptual clarity and future directions. *Int. J. Manag. Rev.* 23 64–84. 10.1111/ijmr.12241

[B95] Wang H. L. Tong L. Takeuchi R. George G. (2016). Corporate social responsibility: an overview and new research directions. *Acad. Manag. J.* 59 534–544. 10.1007/s11356-020-08816-y 32329005

[B96] Wang Y. Jia T. Chen J. Sun H. (2019). Recombine supplier-side search via innovation ambidexterity: an empirical study on Hong Kong manufacturing firms. *Int. J. Phys. Distrib. Logist. Manag.* 49 178–199. 10.1108/ijpdlm-02-2018-0054

[B97] Wang Y. Sun H. Jia T. Chen J. Sun H. (2021). The impact of buyer-supplier interaction on ambidextrous innovation and business performance: the moderating role of competitive environment. *Int. J. Logist. Manag.* 32 673–695.

[B98] Warr P. (2013). “How to think about and measure psychological wellbeing,” in *Research Methods in Occupational Health Psychology, Measurement, Desing and Data Analysis*, eds Wang M. Sinclair R. R. Tetrick L. E. (New York, NY: Routledge), 76–90.

[B99] Wiklund J. Nikolaev B. Shir N. Foo M. D. Bradley S. (2019). Entrepreneurship and well-being. *J. Bus. Ventur.* 34 579–588.

[B100] Wry T. York J. G. (2017). An identity-based approach to social enterprise. *Acad. Manag. Rev.* 42 437–460.

[B101] Xie X. Jia Y. Meng X. Li C. (2017). Corporate social responsibility, customer satisfaction, and financial performance: the moderating effect of the institutional environment in two transition economies. *J. Clean. Prod.* 150 26–39. 10.1016/j.jclepro.2017.02.192

[B102] Ye M. Wang H. Lu W. (2021). Opening the “black box” between corporate social responsibility and financial performance: from a critical review on moderators and mediators to an integrated framework. *J. Clean. Prod.* 313:127919.

[B103] Yu K. Qian C. Zhang L. (2021). Understanding sustainable development flexibility: an information perspective. *Bus. Strategy Environ.* 30 2173–2183. 10.1002/bse.2740

[B104] Yue G. L. Mao S. Z. (2011). Regional differences in entrepreneurship and growth of regional private economy: based on the comparison of private enterprises in Shandong and Zhejiang. *J. Bus. Econ.* 7 43–50.

[B105] Zhang J. A. Edgar F. Geare A. O’kane C. (2016). The interactive effects of entrepreneurial orientation and capability-based HRM on firm performance: the mediating role of innovation ambidexterity. *Ind. Mark. Manag.* 59 131–143. 10.1016/j.indmarman.2016.02.018

[B106] Zhang Y. (1996). The entrepreneurial role of local bureaucracy in China: a case study of Shandong province. *Issues Stud.* 32 89–110.

[B107] Zheng X. Zhu W. Zhao H. Zhang C. (2015). Employee well-being in organizations: theoretical model, scale development, and cross–cultural validation. *J. Organ. Behav.* 36 621–644. 10.1017/S2045796019000787 31839026 PMC8061147

[B108] Zhou D. (2012). Chinese entrepreneurs go global. *Technol. Innov. Manag. Rev.* 2 28–31.

[B109] Zuzul T. Tripsas M. (2020). Start-up inertia versus flexibility: the role of founder identity in a nascent industry. *Adm. Sci. Q.* 65 395–433. 10.1177/0001839219843486

